# Ecophylogenetics Clarifies the Evolutionary Association between Mammals and Their Gut Microbiota

**DOI:** 10.1128/mBio.01348-18

**Published:** 2018-09-11

**Authors:** Christopher A. Gaulke, Holly K. Arnold, Ian R. Humphreys, Steven W. Kembel, James P. O’Dwyer, Thomas J. Sharpton

**Affiliations:** a Department of Microbiology, Oregon State University, Corvallis, Oregon, USA; b College of Veterinary Medicine, Oregon State University, Corvallis, Oregon, USA; c Départment des sciences biologiques, Université du Québec a Montréal, Montreal, Quebec, Canada; d Department of Plant Biology, University of Illinois, Urbana, Urbana, Illinois, USA; e Department of Statistics, Oregon State University, Corvallis, Oregon, USA; University of California, Irvine; Palo Alto Health Care System

**Keywords:** Gut microbiome, bioinformatics, ecology, evolution, phylogeny, taxonomy

## Abstract

Our knowledge of how the gut microbiome relates to mammalian evolution benefits from the identification of gut microbial taxa that are unexpectedly prevalent or unexpectedly conserved across mammals. Such taxa enable experimental determination of the traits needed for such microbes to succeed as gut generalists, as well as those traits that impact mammalian fitness. However, the punctuated resolution of microbial taxonomy may limit our ability to detect conserved gut microbes, especially in cases in which broadly related microbial lineages possess shared traits that drive their apparent ubiquity across mammals. To advance the discovery of conserved mammalian gut microbes, we developed a novel ecophylogenetic approach to taxonomy that groups microbes into taxonomic units based on their shared ancestry and their common distribution across mammals. Applying this approach to previously generated gut microbiome data uncovered monophyletic clades of gut bacteria that are conserved across mammals. It also resolved microbial clades exclusive to and conserved among particular mammalian lineages. Conserved clades often manifest phylogenetic patterns, such as cophylogeny with their host, that indicate that they are subject to selective processes, such as host filtering. Moreover, this analysis identified variation in the rate at which mammals acquire or lose conserved microbial clades and resolved a human-accelerated loss of conserved clades. Collectively, the data from this study reveal mammalian gut microbiota that possess traits linked to mammalian phylogeny, point to the existence of a core set of microbes that comprise the mammalian gut microbiome, and clarify potential evolutionary or ecologic mechanisms driving the gut microbiome’s diversification throughout mammalian evolution.

## Abstract (precis)

Our understanding of mammalian evolution has become microbiome-aware. While emerging research links mammalian biodiversity and the gut microbiome, we lack insight into which microbes potentially impact mammalian evolution. Microbes common to diverse mammalian species may be strong candidates, as their absence in the gut may affect how the microbiome functionally contributes to mammalian physiology to adversely affect fitness. Identifying such conserved gut microbes is thus important to ultimately assessing the microbiome’s potential role in mammalian evolution. To advance their discovery, we developed an approach that identifies ancestrally related groups of microbes that distribute across mammals in a way that indicates their collective conservation. These conserved clades are presumed to have evolved a trait in their ancestor that matters to their distribution across mammals and which has been retained among clade members. We found not only that such clades do exist among mammals but also that they appear to be subject to natural selection and characterize human evolution.

## INTRODUCTION

Research that links the biodiversity of the gut microbiome to mammalian evolution has fueled discussion about the underlying drivers of this relationship (1–5). Perhaps the trillions of microbes inhabiting the gut perform vital functions for their host, such as nutrient acquisition and detoxification (6–8), gut and immune development (9), and pathogen defense (10), that modulate mammalian niche specificity, survival, or fitness. Alternatively, mammalian evolution of physiology, anatomy, behavior, diet, or niche could influence which microbes are exposed to the gut (i.e., the metacommunity) or can thrive within it. However, there remains debate about how potential confounding factors, such as variation in diet (4, 11), management facility (12), or the local metacommunity (13), impact the observed variation of microbiome composition across mammals. The effort to disentangle these and related processes benefits from the identification of specific microbiota that relate to mammalian evolution. For example, studies that uncovered mammalian gut microbiotas that codiversified with their hosts (4, 14) or are subject to vertical inheritance (15, 16) or whose abundance associates with host genotype (16, 17) have supported the idea of the existence of a host genome effect on the gut microbiome.

Understanding how specific gut bacteria distribute across mammalian species can similarly illuminate processes driving the relationship between mammalian evolution and the gut microbiome. For example, microbiotas that are common to mammals may be keystone members of the mammalian microbiome (18), critical to mammalian fitness, and subject to natural selection. Alternatively, these microbes may be apt gut generalists. Additionally, microbiotas that associate with specific mammalian taxonomies may be sensitive to properties derived in the mammalian ancestor, including changes in physiology, diet, behavior, or niche. These properties may impact neutral processes (e.g., random exposure to environmental microbes) or selective processes (e.g., physiological filtering) that influence the microbe’s presence in the gut. These properties may also influence how a mammal’s fitness depends upon the biological functions executed by the microbe. By defining how microbial taxa distribute among mammals, scientists can ultimately zero in on specific taxa to experimentally determine the ecologic and evolutionary processes that drive their association with mammals.

Prior efforts to define this distribution are complicated by the diffuse nature of microbial taxonomy. While the classification of microbes into a Linnaean taxonomy (i.e., phylotyping) detects associations between broad microbial taxonomic categories and host covariates, this approach cannot resolve differences in intermediate levels of taxonomy. Consequently, it fails to identify associations that are complicated by microbial phylogenetic redundancy, wherein multiple phylotypes descended from a common ancestor and share synapomorphic traits that underlie their distribution across hosts. As a result, these phylotypes are functionally interchangeable across hosts and statistical tests that operate at the level of distinct phylotypes may fail to resolve associations due to problems of sparsity. Unfortunately, analyses at higher-order levels of taxonomy do not necessarily solve this problem because of phylogenetic aggregation: higher-order phylotypes include not only the ancestor from which the traits in question derived but also other lineages that do not possess the traits. Consequently, tests of association may fail to detect a signal amidst the noise. These challenges likely confound the resolution of gut microbes that are shared across mammals, given that (i) few enteric microbial operational taxonomic units (OTUs) are shared across mammalian species, while higher-order phylotypes are (1), and (ii) phylogenetic relationships often predict microbial trait conservation (19–21).

**FIG 1:**
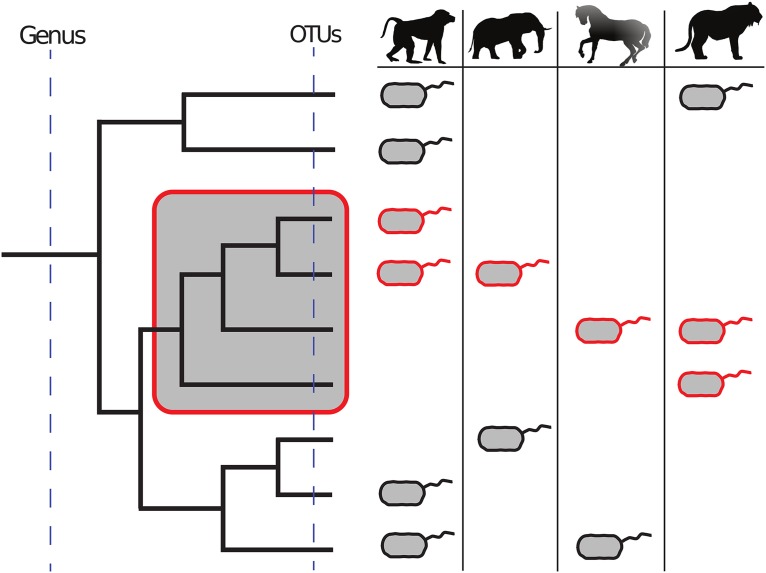
An ecophylogenetic approach to taxonomy can discover ecologically relevant units of microbial taxa. Incorporating phylogeny into the assessment of how microbial lineages distribute across communities can identify monophyletic clades of microbes that collectively manifest an association with ecologic factors or, as in the case of the present study, with host lineages. For example, the clade highlighted in the red-bounded box is universally present across all mammalian microbiome samples, indicating that the ancestor of the clade may have evolved a conserved trait that facilitated its ubiquitous distribution. Considering this relationship at the OTU level (i.e., considering the tips of the tree to be appropriate units) may fail to resolve an association between taxa and their hosts because any member of this clade may possess the trait necessary to occupy the host niche and, consequently, there may not exist clear statistical associations between mammals and this level of taxonomy. On the other hand, if we were to consider the genus level, the aggregation of this clade with other taxonomic groups that do not possess the trait would potentially obscure this relationship.

To circumvent these challenges, we introduce a phylogenetically flexible taxonomy that groups microbial lineages into ecologically relevant taxonomic units (Fig. 1). In particular, we adopt ecophylogenetic theory (22) to identify taxa as monophyletic clades that manifest statistical associations with ecologic covariates. These covariates could include quantitative characteristics, such as environmental pH, or categorical characteristics, such as whether the community was sampled from a marine environment or a terrestrial environment. For example, a clade whose subtending lineages collectively stratify communities in association with a categorical characteristic would represent a taxonomic unit. In the case of the present study, we treated different host lineages as different ecosystems, with the goal of using our approach to resolve microbial clades that statistically associate with a defined set of mammalian lineages.

Our taxonomic approach benefits from several features. First, it leverages phylogenetic relationships, so it is not biased by contemporary taxonomic labels and is not confounded by phylogenetic redundancy or aggregation. In addition, the phylogeny provides a unique opportunity to assess whether a clade’s prevalence across samples is due to chance or not. For example, ancient clades are more likely to contain lineages in a diverse set of communities. Conversely, recently emerged clades might not be found in all communities but might be found in a greater number than would be expected to occur by chance, indicating that a nonrandom process influenced their ecologic diversification. Furthermore, clades that associate with ecologic covariates represent the theoretical evolutionary origin of the microbial traits that underlie the ecologic association. For example, clades that are common across mammals likely derived traits in their ancestor that are critical to the function of the microbial community, the fitness of the host, or the ability of the microbes to disperse and succeed within the host gastrointestinal tract.

We applied this definition of taxonomy to publically available microbiome data spanning diverse mammalian species, with the goal of resolving conserved gut microbiota, which are microbial taxa that are more prevalent across mammalian species than would be expected by chance. Our analysis uncovers clades of gut microbes that are conserved across mammals, as well as clades that are exclusive to and conserved among specific mammalian lineages. These conserved clades were integrated into or lost from mammalian guts in a manner correlated with mammalian evolutionary history. Furthermore, these clades manifest evolutionary patterns consistent with being subject to selection, such as host filtering. These results demonstrate the value of our taxonomic approach, point to the existence of a selectively driven core microbiome of mammals, and broaden the known set of microbes connected to the evolution of mammals.

## RESULTS

### Mammalian evolution associates with conserved clades of bacteria.

We developed an algorithm and corresponding software (clade-based taxonomic units, also known as ClaaTU) that identifies conserved monophyletic clades of taxa, which are clades that display higher prevalence across a set of communities than expected by chance. Briefly, our procedure traverses a phylogeny assembled from 16S rRNA gene sequences generated from multiple communities. It then quantifies each clade’s prevalence across a defined subset of the communities, where the clade’s prevalence is based on the occurrence of the subtending lineages in the subset of communities. A permutation test quantifies whether the observed prevalence of the clade is likely due to chance. While ClaaTU can be applied to any phylogeny, including a sequence-level phylogeny, the following investigations analyzed OTU trees to reduce phylogenetic complexity and subsequently increase statistical power through reduced-numbers hypothesis tests. While sub-OTU patterns may be missed through this heuristic, the fact that few OTUs are shared across host species indicates that sub-OTU variation is unlikely to substantially affect our results.

We used ClaaTU to assess how gut bacterial clades distribute across mammals. We analyzed fecal 16S rRNA gene sequences that were previously generated from 38 individuals spanning 32 mammalian species and 10 orders (1). While these data represent a limited number of individuals, they provide a broad sampling of mammalian phylogenetic diversity. First, we determined that the evolutionary history of mammals associates with the diversity of bacterial clades that comprise their gut microbiome (see Fig. S1 in the supplemental material). We observed a strong and significant association between host order, which served as a proxy for their evolutionary history, and the abundance-weighted (*r*^2^ = 0.44, *P* = 1e−3) or presence-absence (*r*^2^ = 0.57, *P* = 1e−3) Bray-Curtis dissimilarity among gut bacterial communities. Host feeding strategy (carnivory, omnivory, herbivory) and host gut physiology (simple gut, fermentative hindgut, fermentative foregut) more weakly associated with these measures of beta-diversity (feeding strategy abundance-weighted or binary Bray-Curtis, *r*^2^ = 0.26, *P* = 1e−3; gut physiology abundance-weighted Bray-Curtis, *r*^2^ = 0.22, *P* = 1e−3; gut physiology binary Bray-Curtis, *r*^2^ = 0.26, *P* = 1e−3), indicating that they may impact clade diversity but less than host order. That said, the strength of the association of feeding strategy with abundance-weighted beta-diversity appears to be reduced by data corresponding to a subset of omnivores that group more closely with herbivores. Our results indicate that the evolutionary history of the host largely determines which clades are found in the mammalian gut, while the feeding strategy may determine which clades are predominant in the community. These results are consistent with the patterns of phylosymbiosis observed elsewhere (1, 3, 4) but also underscore the importance of diet as a factor determining which taxa dominate the gut microbiome (4, 23).

10.1128/mBio.01348-18.3 FIG S1  The association between microbiome clade beta-diversity, mammalian order, and diet. The levels of abundance-weighted or presence-absence clade Bray-Curtis beta-diversity were quantified between all pairs of mammalian samples and subjected to principal-coordinate analysis for visualization. Permutational multivariate analysis of variance (PERMANOVA) was used to associate beta-diversity with host order or diet. Panel A data represent the clustering of samples by host diet (*r*^2^ = 0.26, *P* = 1e−3) using abundance-weighted beta-diversity. Panel B data represent the clustering of samples by host order (*r*^2^ = 0.44, *P* = 1e−3). The data in panels C (*r*^2^ = 0.26, *P* = 1e−3) and D (*r*^2^ = 0.57, *P* = 1e−3) represent the respective results determined for presence and absence beta-diversity. Download FIG S1, PDF file, 0.4 MB. Copyright © 2018 Gaulke et al. 2018 Gaulke et al. This content is distributed under the terms of the Creative Commons Attribution 4.0 International license.

We then identified clades of bacteria that are conserved across mammals. Of the 8,086 clades harbored by the 38 individuals, 15 were ubiquitous with respect to mammals. However, the false-discovery-rate (FDR [*q* value])-corrected *P* values for these associations were insignificant because these clades appear near the root of the bacterial phylogeny and are thus likely ubiquitous by chance. Future work that considers the prevalence of these clades within a larger framework that includes nonmammalian lineages may reveal that they are indeed conserved within the mammalian gut. Similarly, deeper sequencing and expanded sampling per mammalian lineage may reveal the existence of universal and conserved clades in the mammalian gut. That said, we identified 38 more recently diverged clades that are found in a larger number of mammals than expected by chance (*q* value < 0.2). These conserved clades include members of the class *Alphaproteobacteria*, order *Bacteroidales*, the family *Ruminococcaceae*, and *Prevotella*. The data corresponding to the mammal-conserved *Prevotella* clade (*q* value= 0.04) underscore the potential that conserved clades may be physiologically impactful given that members of *Prevotella* contribute to intestinal health by serving as an energy source for host tissue, regulating inflammation, and promoting motility and blood flow (24).

**FIG 2:**
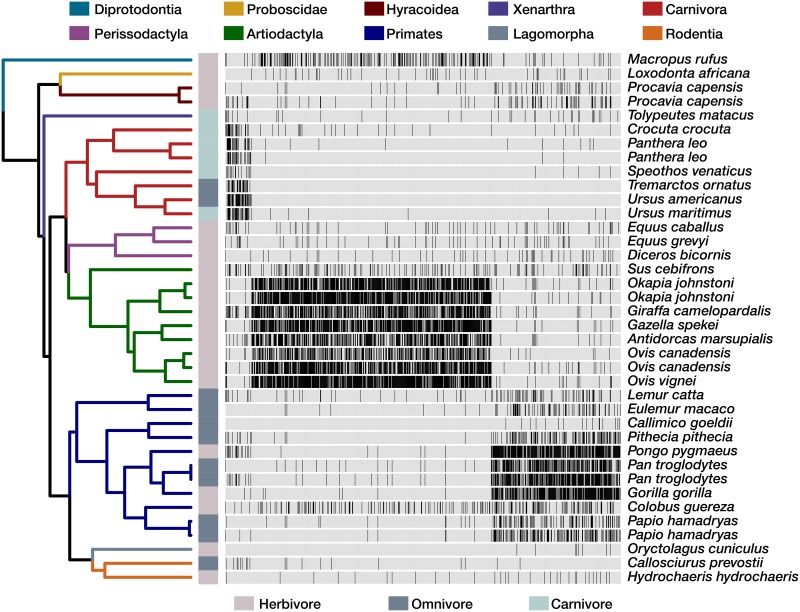
The phylogenetic distribution of conserved bacterial clades reveals associations between gut microbiota and mammalian evolutionary history. The 865 clades that are conserved in at least one mammalian order (*q* value < 0.2) and are not associated with dietary strategy are plotted as columns in a heat map that illustrates their occurrence across mammalian lineages as black ticks. This includes 38 clades that are conserved across the mammals considered in this study. The dendrogram illustrates the evolutionary relationships among mammals, where edges are colored by order and dietary strategy is indicated adjacent to the tips.

We next reasoned that the physiological evolution of mammals could result in filtering or selection for specific clades of gut bacteria. To resolve clades that may relate to the unique physiological aspects of different groups of mammals, we sought to identify clades that are exclusively conserved in distinct mammalian orders (Fig. 2). We constrained this analysis to the Carnivora, Artiodactyla, and Primates, as they were the only orders for which more than three host species were sampled. We identified 322, 591, and 633 clades that were conserved (*q* value < 0.2) in these orders, respectively. Of these, 107, 255, and 245 were also uniquely present in their respective order. For example, all primates included in our study contained a clade of *Prevotella* (*q* value= 6.4e−3) that was also unique to Primates and was consequently distinct from the *Prevotella* clade conserved across mammals. Similarly, a clade within Faecalibacterium prausnitzii was exclusive to and conserved among primates (45% of lineages) included in this study (*q* value= 5.7e−4), which is notable given that underrepresentation of F. prausnitzii in the gut is linked to gastrointestinal disorders (25, 26). Furthermore, a clade within *Turicibacter* was conserved in both Artiodactyla (66% of lineages) and Carnivora (71% of lineages) and was missing from all other hosts. This clade may interact with mammalian traits that evolved in the ancestor of Artiodactyla and Carnivora and that were potentially lost in black rhino, zebras, and horse. In support of this hypothesis, prior work has linked the growth of *Turicibacter* in the gut to host genomic variation (27).

We similarly identified clades that were conserved among mammals that were grouped by dietary strategy to verify that the aforementioned patterns of clade conservation were not due to the potential confounding factor of host dietary strategy (see Text S1 in the supplemental material). In doing so, we corroborated prior work by finding that omnivores carry clades that appear to be specialists with respect to either herbivorous or carnivorous diets (4). However, unlike prior work, we also identified conserved clades of gut bacteria that are unique to omnivores, indicating that omnivore-specialist bacteria may exist.

10.1128/mBio.01348-18.1 TEXT S1  Dietary strategy is associated with conserved clades of gut bacteria. Download TEXT S1, PDF file, 0.03 MB. Copyright © 2018 Gaulke et al. 2018 Gaulke et al. This content is distributed under the terms of the Creative Commons Attribution 4.0 International license.

### Conserved gut microbiota exhibit evolutionary patterns consistent with selection.

We used several phylogenetic methods to discern whether natural selection influences the conservation of bacterial clades across mammals. First, we assessed whether conserved clades are clustered across the bacterial phylogeny; such clustering would indicate that the traits that result in a clade’s conservation could arise through exaptation, be subject to environmental filtering, or improve dispersal (28). We calculated the phylogenetic distances between all pairs of conserved clades and used a Kolmogorov-Smirnov test to determine if the distribution of these distances differs from a bootstrapped distribution of clades randomly sampled from across the same phylogeny. Our analysis uncovered support for the idea of phylogenetic clustering of clades conserved across either Carnivora (*P* = 4.5e−10) or Primates (*P* = 0.03) but not Artiodactyla. Additionally, considering only those clades that are both conserved and unique to Primates (*P* = 0.015), Artiodactyla (*P* = 0.015), or Carnivora (*P* = 2.2e−9) reveals evidence of phylogenetic clustering. We also tested whether clades that are conserved across dietary strategies are clustered, finding that conserved carnivore (*P* = 6.7e−7) and herbivore (*P* = 1.7e−7) clades are clustered, while conserved omnivore clades are not (*P* = 0.11). However, support for clustering was improved by considering only those clades that are both conserved and unique to each of the dietary strategies (carnivore *P* = 2.6e−10; herbivore *P* = 6.7e−7; omnivore *P* = 2.2e−16). We determined that signal propagation of clade conservation among closely related clades did not substantially affect these results by repeating our analysis after excluding conserved clades whose parents were also conserved and finding consistent results. Our observation that conserved clades are phylogenetically clustered indicates that some bacterial lineages are more likely to become conserved than others, possibly because their ancestors evolved a genetic background that potentiated the emergence of traits that improved the fitness of these lineages or resulted in their selection by the host.

**FIG 3:**
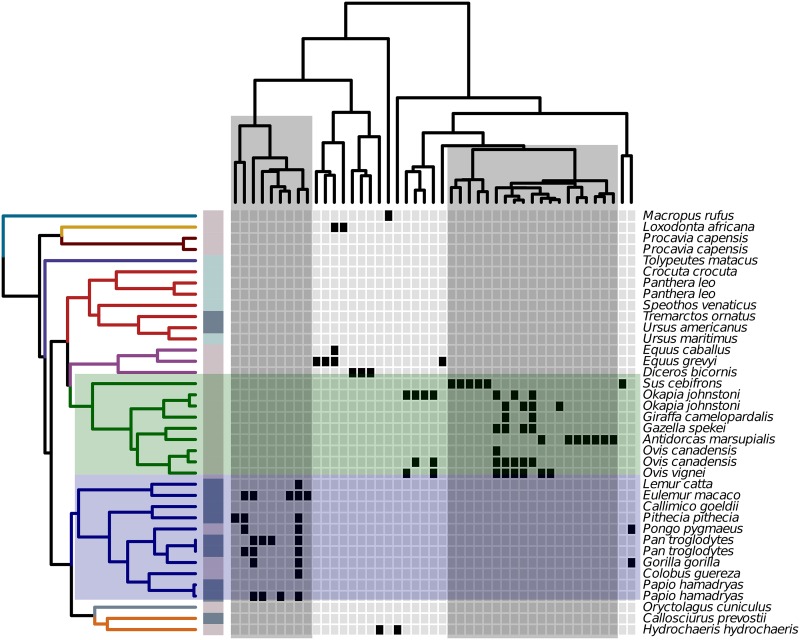
A codiversifying clade within the *Bacteroidales* contains subtending clades that are unique to and conserved among discrete mammalian orders. The evolutionary history of the OTUs in this codiversifying (parafit; *q* < 0.05) bacterial clade is illustrated through the upper portion of the cladogram, while the left-hand cladogram relates mammalian lineages as described in the Fig. 2 legend. Black cells in the heat map indicate that the OTU was detected in a particular individual. Two subclades, highlighted in gray, are conserved among and unique to either the Artiodactyla (green) or Primates (blue).

An emerging body of research indicates that mammals and at least a limited number of their gut microbiota manifest patterns of codiversification (4, 14). We hypothesize that some gut microbiota may be subject to a related process, wherein mammalian evolution associates with the ecologic sorting of bacterial lineages that derived from the same ancestor but which innovated distinct traits that were then subject to selection, phylogenetic redundancy, and conservation among distinct groups of mammals. To explore the existence of such processes, we used parafit (29) to identify 1,171 clades of bacteria that manifest patterns of codiversification with their mammalian hosts (*q* value < 0.05). Of these, 31 clades were conserved in at least one mammalian order, indicating that some clades may become conserved within a group of mammals and subsequently subject to codiversification processes. This includes clades within *Clostridiales*, which prior work found to codiversify with mammals (4), as well as a clade within *Prevotella*. We also found evidence for codiversification of a clade within BS11, which prior work found to be cosmopolitan with respect to ruminants (30), as well as a clade within *Burkholderiales*, which is known to contain lineages that metabolize toxic dietary xenobiotics such as oxalate (31) (Fig. S2). Moreover, we identified 248 clades that codiversified with mammals and were not themselves conserved among any mammalian order but gave rise to multiple descendant clades that were exclusive to and conserved within distinct orders. For example, we identified a clade within the *Bacteroidales* that gave rise to a clade conserved among the Artiodactyla as well as a distinct clade that is conserved among the Primates and annotated as being a member of *Prevotella* (Fig. 3). These results support the hypotheses that at least some anciently integrated members of the mammalian microbiome may have diversified in concert with mammalian evolution and that distinct sets of their descendants can become independently conserved in discrete groups of mammals. Collectively, these findings indicate that the evolution of at least some gut microbiota is linked to the evolution of their mammalian hosts.

10.1128/mBio.01348-18.4 FIG S2  Clades of gut microbiota show evidence of codiversification with their hosts. This figure contains several heat maps that illustrate patterns of codiversification between several clades and their mammalian hosts (parafit; *q* < 0.05). Figures are formatted as described for Fig. 3 of the main text. SF3A (splicing factor 3A), node 100, a member of BS11, which prior work has found to contain cosmopolitan gut microbes (30); SF3B, node 2398, a member of *Burkholderiales*, which contains taxa that prior work has implicated in dietary niche expansion (8); SF3C, node 2401, a codiversifying clade within *Sutterella*; SF3D, node 3138, a clade within *Clostridiales* that manifests a significant signature of codiversification with some mammals according to parafit. Download FIG S2, PDF file, 0.1 MB. Copyright © 2018 Gaulke et al. 2018 Gaulke et al. This content is distributed under the terms of the Creative Commons Attribution 4.0 International license.

### Host lineage-specific variation of conserved clades in hominids.

To clarify how the gut microbiome diversified in association with human evolution (32), we applied ClaaTU to a deeply sequenced set of gut microbiome samples collected from a large number of hominid individuals. Following procedures reported in reference 33, we combined two large 16S rRNA gene fecal microbiome data sets, one consisting of wild chimpanzees (*n* = 146), gorillas (*n* = 177), and bonobos (*n* = 69) and another consisting of humans from the United States (*n* = 314), Venezuela (*n* = 100), and Malawi (*n* = 114), that were prepared using matched molecular methods (34). We assembled these data into a bacterial phylogeny with 69,517 clades. Due to the rate of biological replication found per host lineage, these data afford accurate resolution of lineage-specific features of the microbiome.

**FIG 4:**
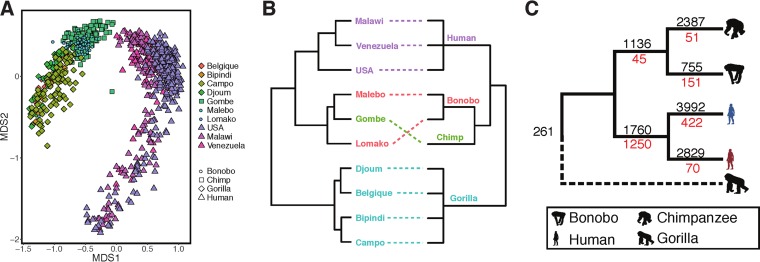
Primate gut microbiomes have diversified in a manner correlated with their evolutionary history. (A) A nonmetric multidimensional scaling plot illustrates the significant differences in clade beta-diversity among primate groups (Adonis; *P* < 0.001). (B) The dendrogram relating groups of primates by their microbiome clade beta-diversity (left) significantly correlates with the phylogenetic distance spanning these same groups (right; Mantel test; *P* < 1e−4). (C) A parsimony imputation of the acquisition (black numbers) and loss (red numbers) of conserved clades among primates that are grouped by their evolutionarily relationships shows that humans have a disproportionately low number of clades that are otherwise conserved among primates and that this effect is amplified in Western humans (blue) compared to non-Western humans (red).

The gut microbiome clade diversity among primates associates with their evolutionary history (Fig. 4) (3). For example, the clade Bray-Curtis dissimilarity stratified individuals based on their host species (Adonis; *R*^2^ = 0.39, *P* < 0.001) as well as on whether they were nonhuman (chimp, bonobo, gorilla) or human (*R*^2^ = 0.28, *P* < 0.001). Furthermore, the clade Bray-Curtis dissimilarity correlated with the phylogenetic distance spanning samples (Mantel test; *R*^2^ = 0.86, *P* < 1e−4). Clade alpha-diversity also varied significantly across species, with notably reduced levels of Shannon entropy in gorillas and Western humans. Collectively, these results corroborate prior work (3) that uncovered evidence of phylosymbiosis among primates and their gut microbiota, despite the fact that our respective studies differed in terms of the host species and individuals sampled in addition to differing with respect to considerations of microbial taxonomy.

We also found that hominid species differ in terms of which bacterial clades are conserved in their microbiomes. We identified 18,942 clades that were conserved (FDR < 0.01) in at least one group of hominids (chimpanzee, gorilla, bonobo, Western human, non-Western human), only 261 of which were ubiquitously conserved (Fig. S3). An additional 1,250 were conserved among the wild apes, while 1,760 clades were conserved in Western and non-Western humans. Of these, 265 and 352 clades were exclusive (i.e., unobserved outside the group) to nonhuman primates and humans, respectively. For example, 66 clades associated with the short-chain-fatty-acid-producing family *Lachnospiraceae* were exclusively conserved in humans and absent in the other primates (35). Nonhuman hominids contained 43 exclusively conserved clades associated with the polyphenol-metabolizing family *Coriobacteriaceae* (36). Western humans had the greatest number of conserved as well as exclusive and conserved clades. They also harbored the highest fraction (55%) of clades that were uniquely conserved (i.e., present in other lineages but not conserved). These results indicate that Western humans share substantial portions of their microbiome that are distinct from those of non-Western humans and the nonhuman primates, including 508 conserved clades from the genus *Bacteroides*, 107 from *Ruminococcus*, and 54 from *Akkermansia*. These results agree with previous reports that indicate that diets rich in fats, such as the standard Western diet, support microbiomes high in *Bacteroides* (37). Conversely, non-Western individuals harbored a large number of uniquely conserved clades from the genera *Prevotella* (733 clades), *Streptococcus* (79 clades), *Lactobacillus* (57 clades), and *Bifidobacterium* (56 clades). Chimpanzees, bonobos, and gorillas also harbored several clades associated with the genus *Prevotella* (128, 80, and 63 clades, respectively), while Western humans contained only 3, indicating that substantial conserved clade diversity of *Prevotella* is a feature missing from Western humans. Our results align with previous observations that *Prevotella* abundance associates with dietary fiber intake (37) and further suggest that consumption of low-fiber diets, such as the standard Western diet, may result in decreased richness of *Prevotella* and increased richness of *Bacteroides* clades in the gut. Collectively, this analysis indicates that changes in lifestyle, environment, or genetics that associate with Westernization cooccur with changes to the suite of conserved bacteria that occupy the gut.

10.1128/mBio.01348-18.5 FIG S3  Primates share substantial clade diversity. A Venn diagram illustrates the number of conserved clades that are shared among primate groups and the number that are unique to each primate group. Download FIG S3, PDF file, 0.2 MB. Copyright © 2018 Gaulke et al. 2018 Gaulke et al. This content is distributed under the terms of the Creative Commons Attribution 4.0 International license.

Given these patterns of clade conservation across hominids, we used a parsimony approach to identify gut bacterial clades that have become conserved (i.e., gains) or that are no longer conserved (i.e., losses) along specific hominid lineages. For this analysis, gorilla was used as an out-group, which prevented the assessment of gains and losses along this lineage. We observed a relatively extensive gain of conserved clades by each species during the course of hominid evolution (Fig. 4). Gains in all lineages outpaced losses potentially due to additional niches opening in the gut during speciation, dietary transition, or altered habitat.

The human gut microbiome dramatically differs from those of other hominids in terms of conserved clades. For example, humans lost a disproportionately large number of hominid conserved clades. Westernized humans exemplify this trend. These clades include members of common human gut genera, such as *Prevotella* (45 clades), *Methanobrevibacter* (19 clades), and *Bifidobacterium* (4 clades) (Fig. S4). Humans also gained conserved clades associated with the genera *Bacteroides* (64 clades), *Bifidobacterium* (32 clades), and *Ruminococcus* (26 clades). These results agree with prior research that indicated that the abundance of *Ruminococcus*, *Bifidobacterium*, and *Bacteroides* differentiated nonhuman from human primates (33). Our findings suggest that the evolution of humans is linked to substantial alteration of the factors that impact gut microbiome clade conservation. While the underlying processes driving these patterns are not known, they potentially include (i) interlineage variation in ecology and environment, including differences in shelter and sanitation that could affect microbial metacommunity exposure (38); (ii) genomic evolution, as genotype-microbiome interactions have been described in humans (17); (iii) aspects of behavior or diet that may influence microbial dispersal and growth in the gut; and (iv) cryptic study effects that bias the resolution of microbial lineages in specific host groups. An expanded sampling of primate lineages, across diverse populations, coupled with rich metadata would help determine whether these processes contribute to clade conservation in primates.

10.1128/mBio.01348-18.6 FIG S4  A clade within *Clostridiales* is conserved across primates, but its descendants are differentially conserved across host groups. This cladogram of node 188 is annotated with pie charts on internal nodes that illustrate which groups of primates the clade is conserved among. For example, a green circle indicates that the clade is exclusively conserved among chmips, while a circle with red, blue, green, purple, and orange wedges indicates that the clade is conserved in every primate group. Of particular note is the clade highlighted with a red arrow, which is conserved among all nongorilla groups but whose descendants are either exclusively conserved in Western humans or are conserved in all nongorilla groups other than Western humans. This ecophylogenetic pattern illustrates how processes such as lineage sorting may be giving rise to the patterns of gains and losses of conserved clades described in the main text. Download FIG S4, PDF file, 0.2 MB. Copyright © 2018 Gaulke et al. 2018 Gaulke et al. This content is distributed under the terms of the Creative Commons Attribution 4.0 International license.

## DISCUSSION

Our ecophylogenetic approach offers a new way to uncover discoveries in the study of microbial communities. Determining the appropriate taxonomic rank at which to analyze microbial community data presents a key challenge to investigators. Ranks that are too granular or too broad can overpartition statistical signal or overwhelm it with a level of noise sufficient to eliminate the ability to detect meaningful patterns. To mitigate this problem, our approach integrates across a phylogeny to resolve monophyletic clades at any rank that associate with study (ecologic) covariates, which in the case of the present study included the specific set of mammalian hosts that carried clade members. A key innovation of our approach is the ability to use the phylogenetic tree’s topology to quantify whether the observed associations are likely to have arisen due to chance given the phylogenetic breadth of the clade in question. In the case of the discoveries presented here, this feature enabled the detection of microbial taxa whose nonrandom prevalence among mammals indicates their conservation. Moreover, because our approach relies on detecting statistical patterns that associate with monophyletic clades, it offers a theoretical point of origin for the traits that drive the clade’s distribution across the studied ecosystems (i.e., the common ancestor). While we analyzed a phylogeny assembled from OTU-clustered 16S rRNA gene sequences, our approach is agnostic with respect to the specific tree construction procedure or phylogenetic markers being analyzed. Our method extends prior studies that transformed characterization of microbial communities through consideration of phylogeny (39–44) and ultimately helps pave the way for phylogenetically informative definitions of microbial taxonomy (45).

To our knowledge, our approach is the first to resolve microbial taxa that are more prevalent across mammals than expected by chance and points to the existence of a core suite of phylogenetic clades that define the mammalian gut microbiome. We were unable to ascertain the drivers of this apparent conservation of gut microbiota; these conserved bacterial clades may represent (i) gut generalists that are effective at dispersal or (ii) taxa that elicit a beneficial effect for their mammalian host and consequently have been retained by mammals throughout their evolution due to natural selection. Nonetheless, identification of these conserved clades of gut microbiota is important because the data would clarify which microorganisms researchers should experimentally interrogate to discern the mechanisms that influence the ecologic and evolutionary diversification of the mammalian gut microbiome. For example, future work can ascertain whether natural selection influences the distribution of these microbial taxa across mammals (18) and ultimately resolve the specific biological traits subject to selection.

Moreover, efforts to engineer probiotic gut communities benefit from the identification of conserved clades, as such clades may be required for successfully maintaining homeostasis in the gut. That said, relatively few clades were present in all mammalian lineages, and most of the conserved clades identified were not fully present across all individuals within a considered group. This pattern might arise as a result of convergent evolution, wherein disparate lineages of gut bacteria independently evolve an ecologically selected trait, such as the presence of glycosyl hydrolases (46). Alternatively, mammalian evolution may have yielded changes in the selective regime for specific microbiome traits. Such changes would reduce the ubiquity of these clades across mammals. Future work should consider expanding the numbers of individuals sampled from each host lineage to improve the resolution of species-specific associations with the microbiome, especially given that enterotypic variation (2) or low depth of sampling could create the signature of a clade’s absence when few individuals are interrogated within a host species. Indeed, in our expanded analysis of hominids, we resolved the complete absence of some clades in humans that are otherwise conserved across hominids despite an extensive sampling of individuals. These findings support the hypothesis that there exist host lineage-specific dependencies on the gut microbiome but would benefit from further work that controls for management facility, geographic, and dietary effects.

Our analysis considered only bacteria from stool and did not consider the environmental metacommunity from which animals sample their microbiomes (13). This matters for several reasons. First, the metacommunity may have changed over the course of mammalian evolution. Thus, our findings do not necessarily indicate that the microbiome has codiversified with the hosts or even that the observations made here reflect conditions that were necessarily present throughout the evolution of the various lineages being studied. Indeed, it is possible that these clades are transient or were environmentally acquired, especially since many of these animals were kept in captivity and potentially in close proximity and because relatively few individuals were analyzed per host species in some of our analyses. However, given the phylogenetic patterns in our data, we can expect that the traits that make contemporary microbes successful at colonizing a large number of hosts were inherited from those bacteria that successfully colonized ancestral hosts. Second, it is possible that metacommunity variation contributes to the observed differences between taxa. For example, the clades that stratify humans from nonhuman primates are perhaps not ubiquitous among the metacommunities of these two sets of hosts. Consequently, the conserved clades that stratify hosts may do so because they possess traits that (i) enable their success in their respective metacommunities and (ii) enable their frequent migration into the guts of their respective hosts. Third, while there appears to be some evidence of inheritance in our results, we note that this does not necessarily indicate that direct, vertical transmission of microbiota between generations has occurred. Indeed, patterns of microbiome inheritance may result from vertical transmission of host genotype that selects for specific metacommunity assemblages. Future work should deeply sample wildlife populations as well as their microbial metacommunities to experimentally discern the mechanisms underlying the high prevalence of these clades of gut bacteria among mammals. For example, longitudinal sampling of wildlife populations can clarify the migration rates and stability of clades in the mammalian gut.

The fact that gut microbiotas contribute to mammalian health and behavior motivates efforts to identify gut microbes that influence mammalian fitness or even coevolve with mammals (1, 5, 33, 47). Our finding that conserved clades of gut bacteria phylogenetically sort among mammalian orders and primate species aligns with prior work that links the evolution of mammals to the diversification of their microbiomes (1, 3, 4, 14). However, the underlying driver of this association (e.g., coevolution) remains unclear. Our analytic approach offers a unique theoretical benefit in the effort to resolve the basis for these associations: it integrates the phylogeny of the hosts alongside the phylogeny of their gut microbes, which can clarify the respective orders of host and microbial diversification. For example, our observation of phylogenetic sorting would support a hypothesis of coevolution if the date at which microbial clades diversified cooccurred with the date at which their corresponding mammalian hosts radiated (48). Conversely, we might favor a model wherein selection acts to filter the pool of microbes that contact the host’s gut if the microbial clades in question emerged prior to the corresponding mammalian lineages. Unfortunately, our study’s reliance on 16S rRNA gene sequence data complicates efforts to date microbial divergence times, given that 16S rRNA gene mutation rates can differ greatly across different bacterial lineages, which in turn yields inaccurate divergence time estimates (49, 50). Moreover, while analysis of the 16S rRNA gene locus offers tremendous benefits for microbiome research, its relatively low mutation rate may prevent resolution of cryptic divergences and may thus impact the interpretation of any such test of synchrony. The latter point is especially important with respect to considerations of microbial divergences among recently derived mammalian lineages (14). Researchers performing future work should instead endeavor to leverage phylogenies assembled from shotgun metagenomes (51), where strain-level resolution (52) and genome-wide rates of evolution (53) are discernible. Alternatively, studies should employ the use of universal marker genes that offer more-pronounced insight into evolutionary rates, such as the gyrase B gene (14). Finally, efforts to define local molecular clocks for specific microbial clades, which tend to offer improved accuracy (50), can facilitate clade-specific analyses of synchronous divergence. By applying our approach to the data constructs detailed above, we may ultimately resolve candidate microbial taxa that coevolve with their mammalian hosts. Our investigation aligns with recent efforts to ascribe periods of microbial evolution to the emergence of specific traits (21, 42, 54). In particular, our informatics procedure identified monophyletic clades that contain phylogenetically redundant lineages, indicating that the taxa that comprise the clade contain a trait that arose in the clade’s ancestor or at a prior developmental stage. However, limitations in the phylogenetic breadth of the data analyzed here may result in inaccurate imputation of a trait’s evolutionary origin. Future work should seek to map traits onto comprehensive phylogenies to assess when traits arose and which specific traits are likely contributing to clade conservation. Doing so will consequently facilitate empirical investigations of the role of these traits in the operation of the gut microbiome, microbial dispersal, and host fitness.

## MATERIALS AND METHODS

### Identification of ecophylogenetic taxonomic units.

We developed ClaaTU (https://github.com/chrisgaulke/Claatu), an algorithm that quantifies the abundance of monophyletic clades of taxa across a set of communities and optionally identifies clades that are more prevalent than expected by chance. ClaaTU conducts a brute force root-to-tip traversal of a phylogenetic tree and quantifies the abundance of each monophyletic clade in each community by summing the abundances of subtending lineages. To determine if a clade is conserved, ClaaTU converts abundances to presence-absence data and then quantifies each clade’s prevalence across a set of samples. To ascertain if the observed prevalence is greater than that expected by chance, ClaaTU conducts a phylogenetic permutation test (44). Specifically, the observed phylogenetic tip-to-community labels are randomly shuffled, such that the underlying prevalence distribution remains fixed, while the associated lineages are altered. This random permutation of the data occurs multiple times (1,000 times for the mammal-wide study and 100 times for the primate study due to limitations of tree size) to produce a bootstrapped prevalence distribution for each clade. A *z* test determines if the clade’s observed prevalence is significantly greater than the bootstrapped null distribution. ClaaTU assigns a taxonomic label to each clade by identifying the most granular taxonomic assignment shared by all subtending lineages.

### Analysis of mammalian microbiome samples.

We analyzed publicly available data. First, quality-controlled 454 pyrosequenced reads generated from the V2 region of the 16S rRNA gene of stool samples collected from 31 animals representing 10 taxonomic orders (1) were downloaded from MG-RAST (accession no. mgp113 and mgp114). Reads were clustered into operational taxonomic units (OTUs) using pick_open_reference_otus.py in QIIME (55) with UCLUST (56) and a 97% identity threshold against the greengenes database (13_8). Taxonomy was inferred for each OTU using assign_taxonomy.py in QIIME with default parameters.

Second, a data set of region V4 16S rRNA gene sequences generated from stool collected from 146 wild chimpanzees, 69 bonobos, and 177 gorillas from several field sites (33) was downloaded from http://web.biosci.utexas.edu/ochman/moeller_data.html and filtered to trim low-quality bases (*q* value < 25) using split_libraries.py in QIIME. A separate data set of region V4 16S rRNA gene sequences (34) consisting of 528 human sequences—314 of which were from the United States (Western) and 114 and 100 of which were from Malawi and Venezuela, respectively (non-Western)—was downloaded from MG-RAST (accession no. mgp401). Following prior work (33), the nonhuman and human data sets were combined, trimmed to a uniform sequence length of 99 bp, and collectively processed. OTU clustering and taxonomic annotation occurred as described above. The resulting data set was filtered to remove low-frequency OTUs (present in fewer than nine samples) and low-abundance OTUs (total abundance of <10 counts). Using a subset of hominid samples, we determined that the phylogeny produced using QIIME-clustered OTUs highly correlates with a corresponding phylogeny assembled using DADA2, indicating that the results produced using an OTU clustered phylogeny are indicative of those obtained using an exact sequence variance approach (see Text S2 in the supplemental material).

10.1128/mBio.01348-18.2 TEXT S2  Phylogenies assembled from OTU clustered sequences correlate with those assembled using exact sequence variants. Download TEXT S2, PDF file, 1.4 MB. Copyright © 2018 Gaulke et al. 2018 Gaulke et al. This content is distributed under the terms of the Creative Commons Attribution 4.0 International license.

### Tree construction and clade diversity quantification.

The QIIME-assigned OTU representative sequences were used to assemble phylogenetic trees for each data set. In the case of the primate data, these sequences were combined with the greengenes 97% identity set of full-length reference sequences to improve the phylogenetic accuracy of short-sequence data, following methods described in references 57 and 58. Infernal (59) was used to align sequences as described in references 57 and 58. Alignment columns containing 50% gap characters were removed using filter_alignment.py in QIIME. FastTree was employed to construct phylogenies using a generalized time-reversible model (60). Phylogenies were pruned of greengenes reference sequences and subjected to midpoint rooting and processing by ClaaTU.

### Statistical analyses.

Statistical analyses were conducted using R. Bray-Curtis dissimilarity was calculated using vegan::vegdist, while vegan::diversity was used to quantify Shannon entropy. Permutational multivariate analysis of variance was used to quantify the association between beta-diversity and categorical sample covariates (vegan::Adonis; 1,000 permutations). To determine if the patterns of microbiome diversity were similar to the evolutionary history of primates, the Bray-Curtis disimilarity matrix was correlated to the phylogenetic distance matrix spanning all pairs of samples using the Mantel function with 1,000 permutations. The hominid phylogeny was obtained from the 10kTrees (version 3) website (https://10ktrees.nunn-lab.org) (61). For all procedures, false-discovery-rate analyses were used to correct multiple tests. All related analytic software and results can be found at http://files.cgrb.oregonstate.edu/Sharpton_Lab/Papers/Gaulke_mBio_2018/.
