# Supplementary figures and images for: Ecophylogenetics Clarifies the Evolutionary Association between Mammals and Their Gut Microbiota
Source: mBio. 2018 Sep 11;9(5):e01348-18. doi: 10.1128/mBio.01348-18 (PMC6134092; doi:10.1128/mBio.01348-18)

A

Diet Abundance

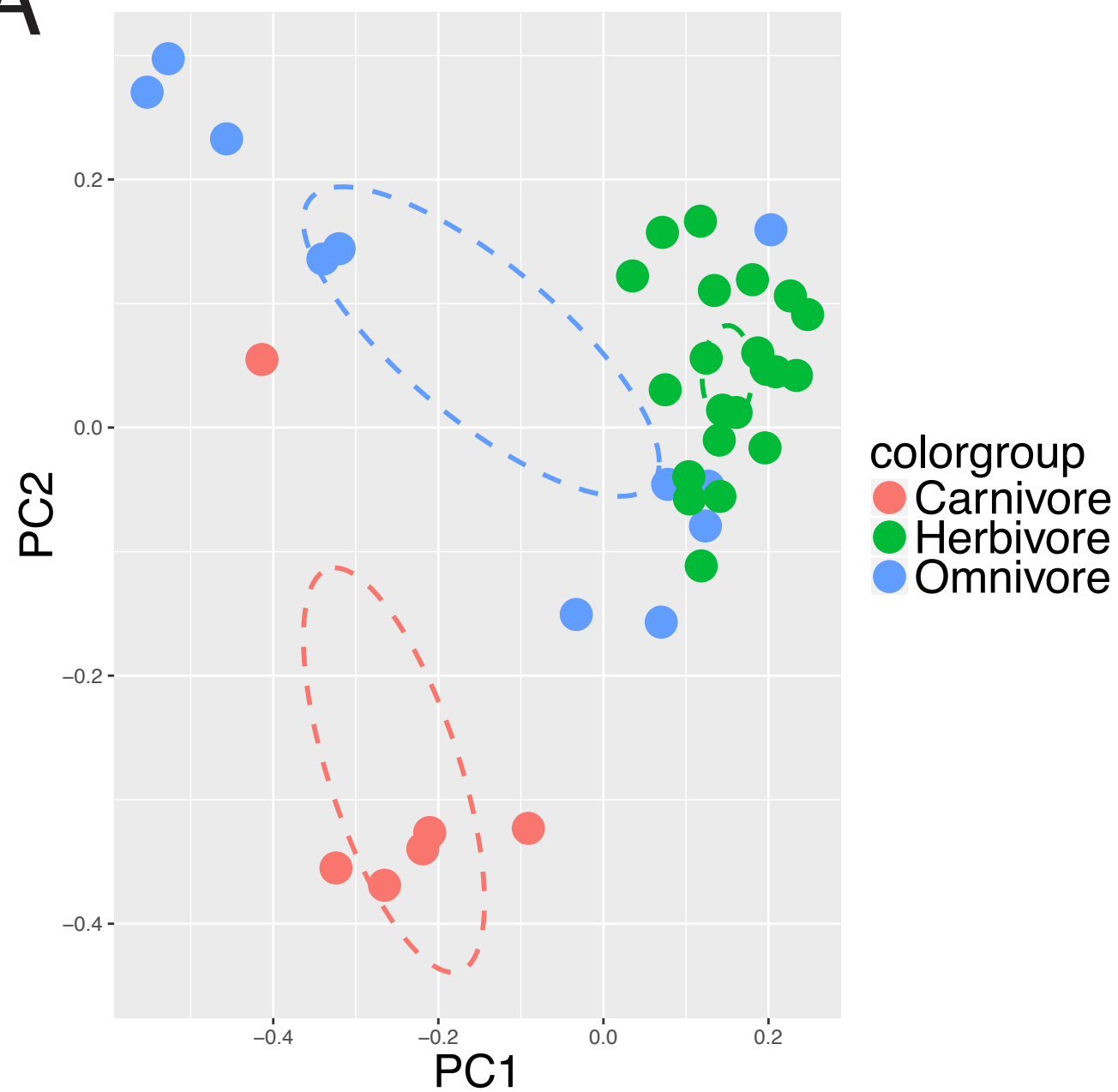

B

Order Abundance

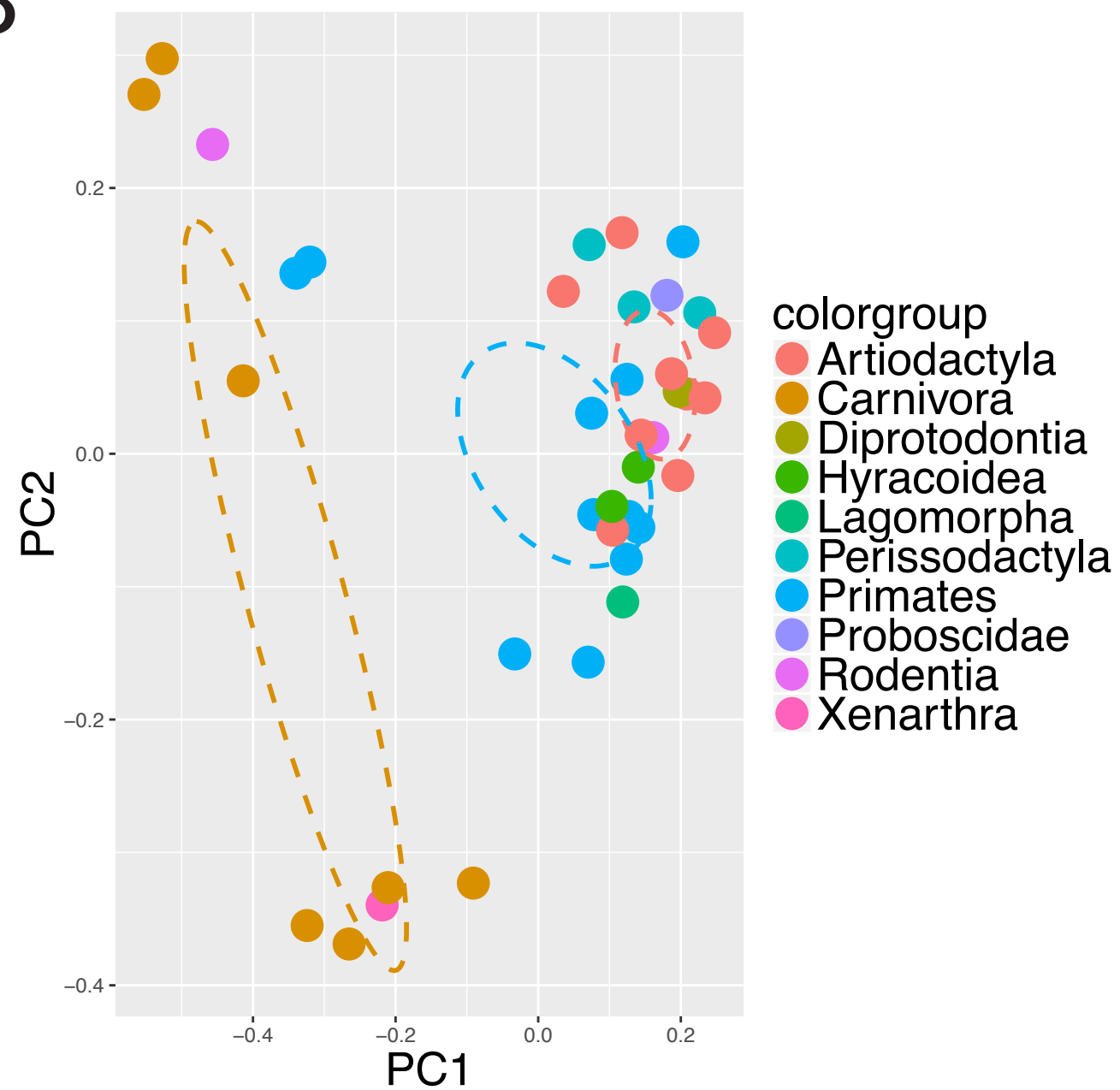

C

Diet Presence Absence

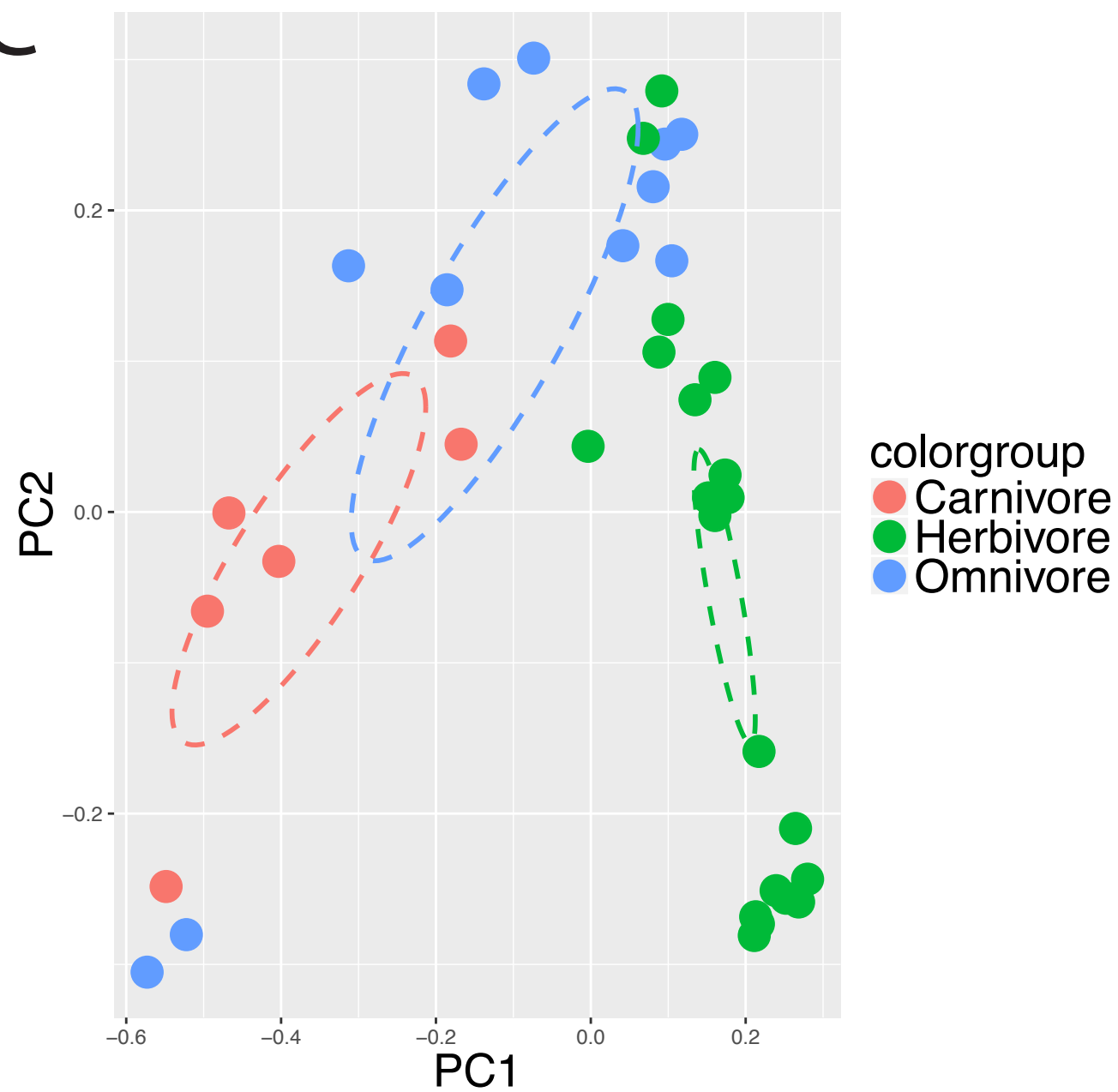

D

Order Presence Absence

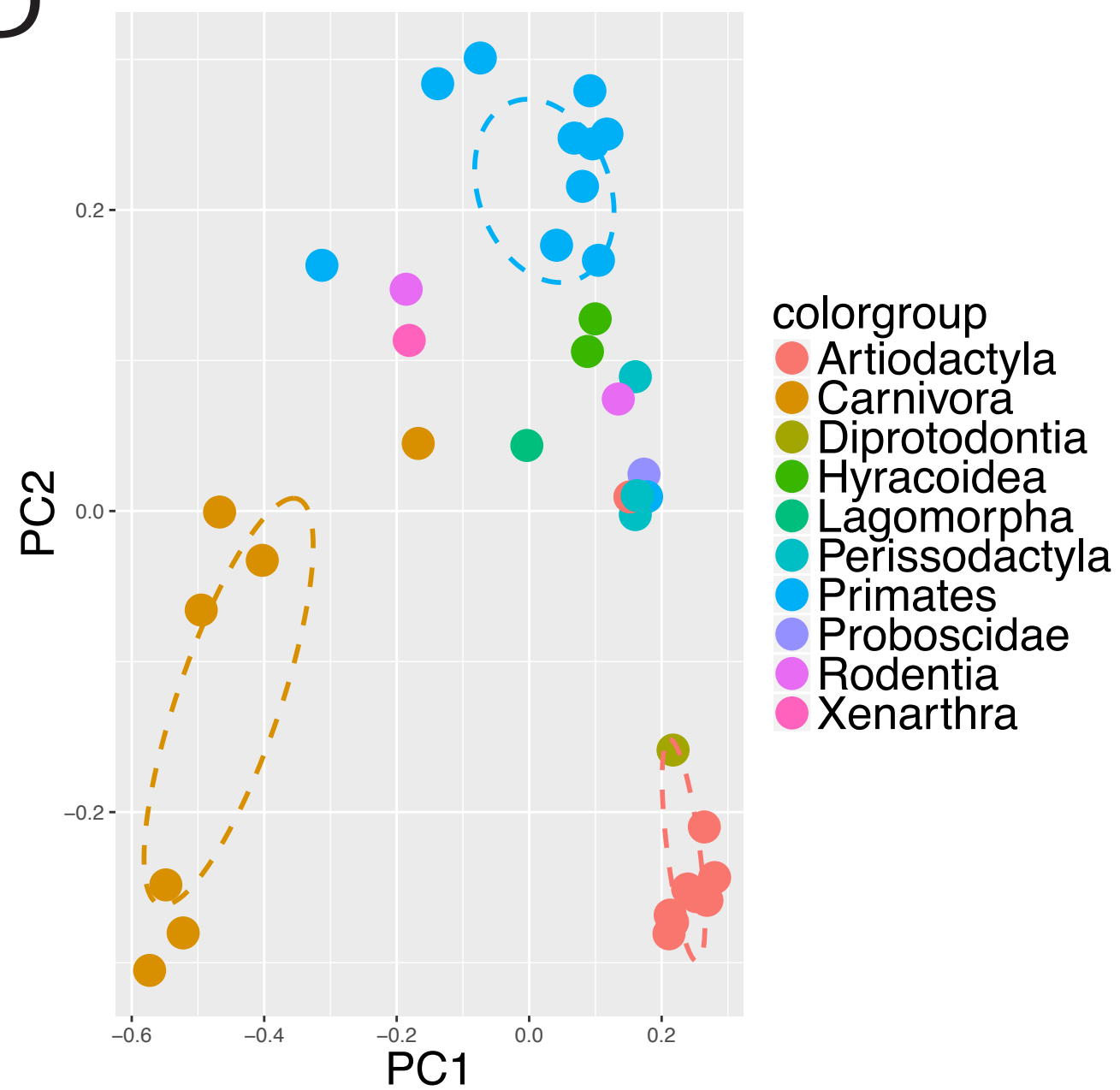

Supplement: FIG S1 [file mbo004184055sf1.pdf]

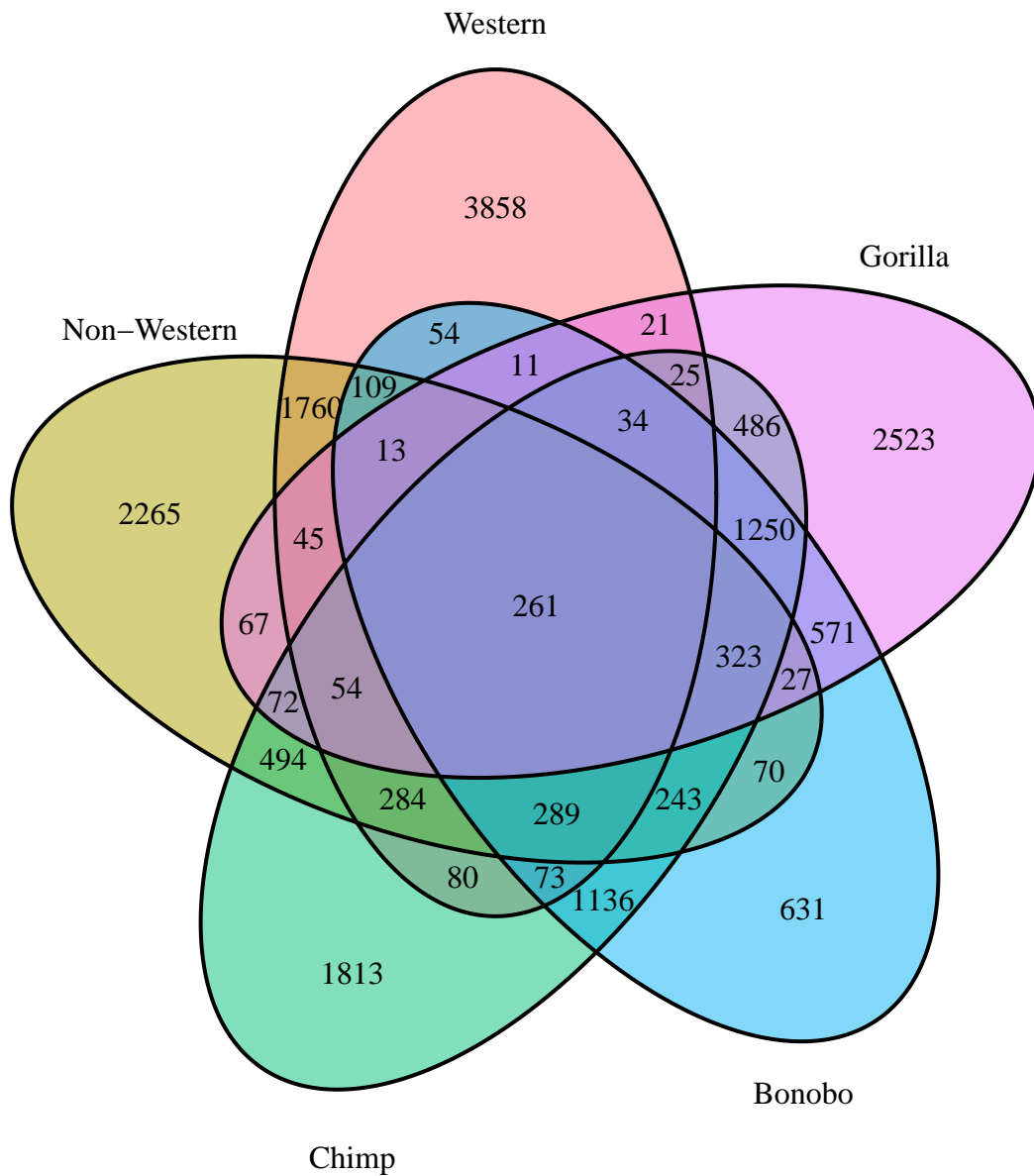

Supplement: FIG S3 [file mbo004184055sf3.pdf]

# node188

- western
- non\_western
- chimp
- bonobo
- gorilla

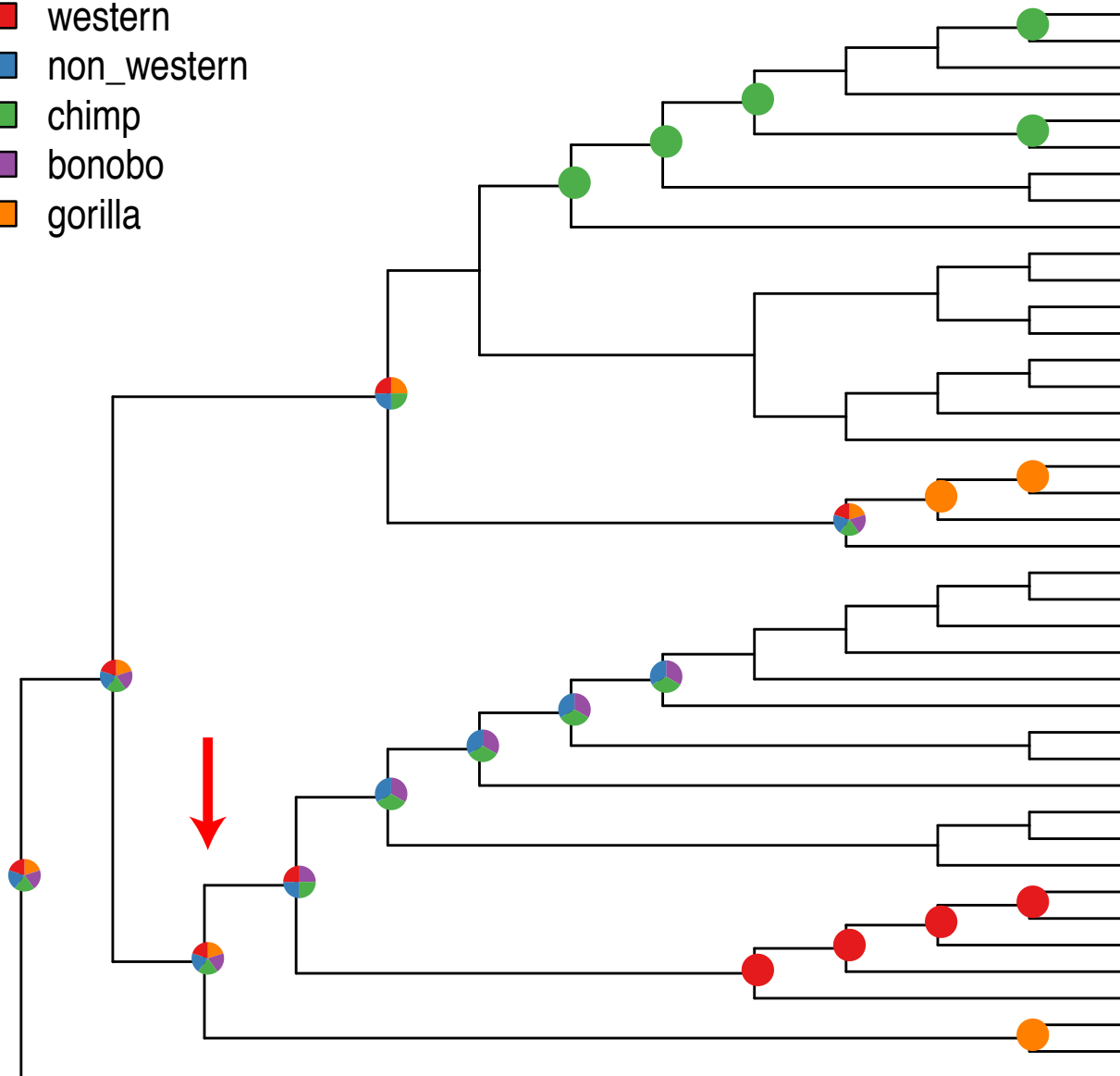

Supplement: FIG S4 [file mbo004184055sf4.pdf]
