# Supplementary material for: Ecophylogenetics Clarifies the Evolutionary Association between Mammals and Their Gut Microbiota
Source: mBio. 2018 Sep 11;9(5):e01348-18. doi: 10.1128/mBio.01348-18 (PMC6134092; doi:10.1128/mBio.01348-18)
Supplement: TEXT S1 [file mbo004184055s1.pdf]

Supplemental Text: Dietary strategy is associated with conserved clades of gut bacteria

The gut microbiome plays a critical role in digestion and enables the host to access many dietary nutrients. We postulated that there exist clades of gut bacteria that express traits that are associated with mammalian nutritional strategy. For example, clades that are conserved across and unique to herbivores may be dietary specialists and are potentially critical to an herbivore's nutritional success. To investigate nutritional specialization, we used ClaaTU to identify clades that are conserved across herbivores, carnivores, or omnivores. Doing so identified 750 clades that were significantly associated with at least one category of host dietary strategy, 158 of which were also unique to a dietary strategy. Specifically, we identified 364, 63, and 334 clades that were conserved among omnivores, herbivores, and carnivores, respectively, 21, 43, and 94 of which were also unique to the dietary category in which the clade is conserved. Interestingly, omnivores harbor a larger number of conserved clades than herbivores and carnivores, though the vast majority of these clades are not unique to omnivores. Indeed, of the conserved omnivore clades, 335 occur in at least one herbivore and 76 occur in at least one carnivore, though only 8 of these are exclusive to carnivores and omnivores. These results are generally consistent with prior work that concluded that omnivores harbor gut bacteria that are specialists to herbivorous or carnivorous diets (1). However, unlike prior work, we also identify lineages of gut bacteria that appear to be specialists to the omnivorous diet. This includes a clade within the genus *Blautia,* which is a genus present in the human gut that was found to increase in abundance when patients consumed diverse and varied diets that include whole grains (2), and is within the Clostridium XIVa cluster (3), which was found to associate with omnivory (4). These conserved clades that are unique to omnivores may express traits that make them apt at growing on the diverse nutrient resources consumed by omnivores. This could be an important trait for their hosts, as the efficient digestion of food requires gut microbial metabolism and changing diets results in lag in the growth of bacteria that can metabolize the substrate (5). Turnbaugh}. Consequently, dietary generalist gut microbes may help omnivores efficiently extract nutrients from their varied diet. In contrast, we identified relatively few conserved clades among the herbivores, though most of them were unique to herbivores. This may indicate that herbivory is not associated with frequent gut microbe specialization, possibly because omnivores frequently consume similar diets and thus select for similar microbial traits, or that convergent evolution and functional redundancy among gut bacteria obscures the detection of such specialization. However, those gut bacteria that are strongly associated with herbivory tend to be exclusive to herbivores, indicating that there are herbivore-specific properties that drive the conservation of these clades. Finally, carnivores harbor the largest number of conserved and unique clades of gut bacteria, even if omnivores are treated as herbivores for the purpose of considering clade uniqueness, indicating that dietary specialization has occurred relatively frequently among the gut bacteria that associate with carnivores. We note that dietary strategy may be confounded with the transit time of the bolus through the gastrointestinal tract and consequently some of the associations with dietary strategy could instead be associated with the amount of time that the gut bacteria have to grow on the nutrients available in the bolus.

1. Groussin M, et al. (2017) Unraveling the processes shaping mammalian gut microbiomes over evolutionary time. *Nat Commun* 8:14319.
2. Martínez I, et al. (2013) Gut microbiome composition is linked to whole grain-induced immunological improvements. *ISME J* 7(2):269–280.
3. Eren AM, et al. (2015) A single genus in the gut microbiome reflects host preference and specificity. *ISME J* 9(1):90–100.
4. Kabeerdoss J, Shobana Devi R, Regina Mary R, Ramakrishna BS (2012) Faecal microbiota composition in vegetarians: comparison with omnivores in a cohort of young women in southern India. *Br J Nutr* 108(6):953–957.
5. Turnbaugh PJ, Bäckhed F, Fulton L, Gordon JI (2008) Diet-induced obesity is linked to marked but reversible alterations in the mouse distal gut microbiome. *Cell Host Microbe* 3(4):213–23.
