# Supplementary material for: Ecophylogenetics Clarifies the Evolutionary Association between Mammals and Their Gut Microbiota
Source: mBio. 2018 Sep 11;9(5):e01348-18. doi: 10.1128/mBio.01348-18 (PMC6134092; doi:10.1128/mBio.01348-18)
Supplement: FIG S2 [file mbo004184055sf2.pdf]

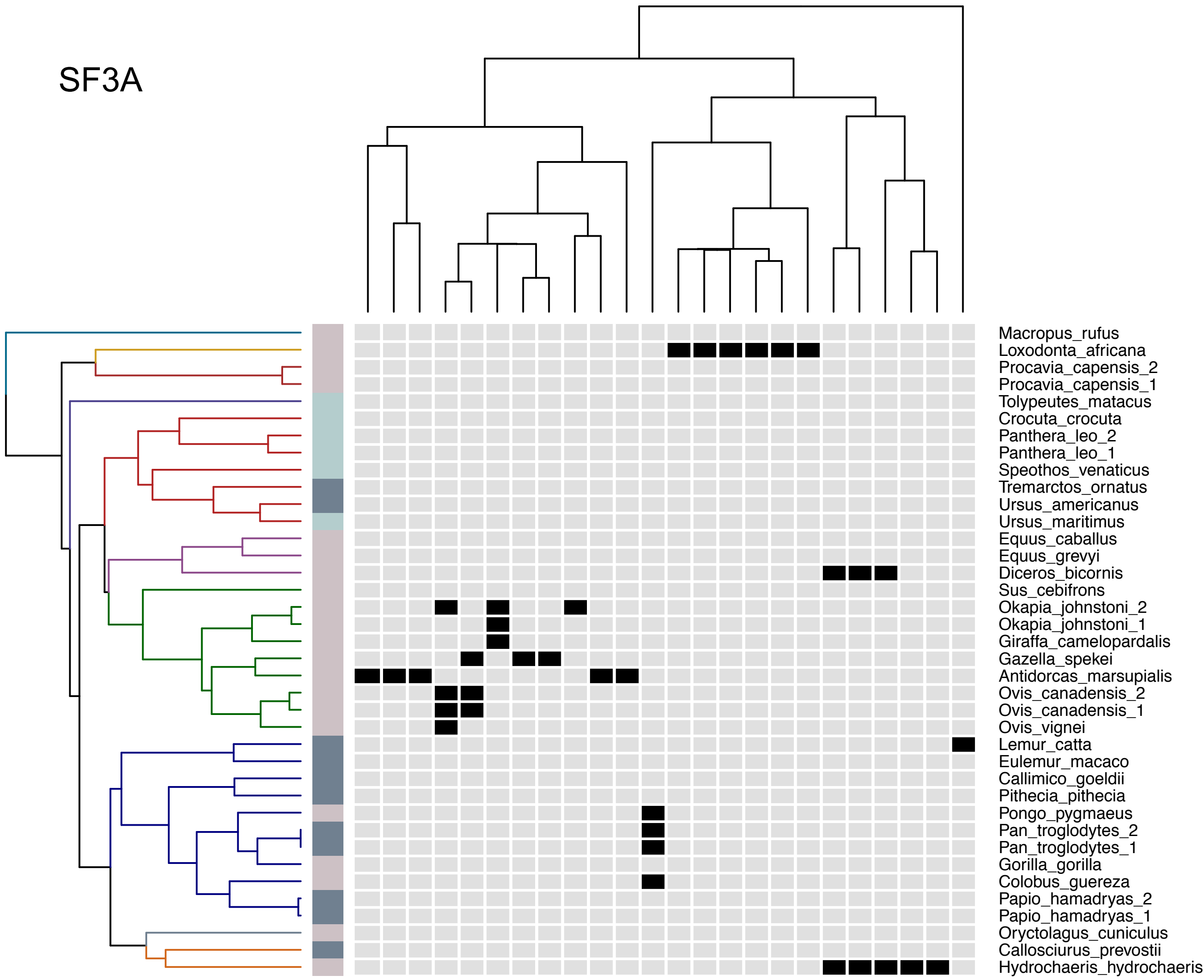

SF3A
Macropus_rufus
Loxodonta_africana
Procavia_capensis_2
Procavia_capensis_1
Tolypeutes_matacus
Crocuta_crocuta
Panthera_leo_2
Panthera_leo_1
Speothos_venaticus
Tremarctos_ornatus
Ursus_americanus
Ursus_maritimus
Equus_caballus
Equus_grevyi
Diceros_bicornis
Sus_cebifrons
Okapia_johnstoni_2
Okapia_johnstoni_1
Giraffa_camelopardalis
Gazella_spekei
Antidorcas_marsupialis
Ovis_canadensis_2
Ovis_canadensis_1
Ovis_vignei
Lemur_catta
Eulemur_macaco
Callimico_goeldii
Pithecia_pithecia
Pongo_pygmaeus
Pan_troglodytes_2
Pan_troglodytes_1
Gorilla_gorilla
Colobus_guereza
Papio_hamadryas_2
Papio_hamadryas_1
Oryctolagus_cuniculus
Callosciurus_prevostii
Hydrochaeris_hydrochaeris

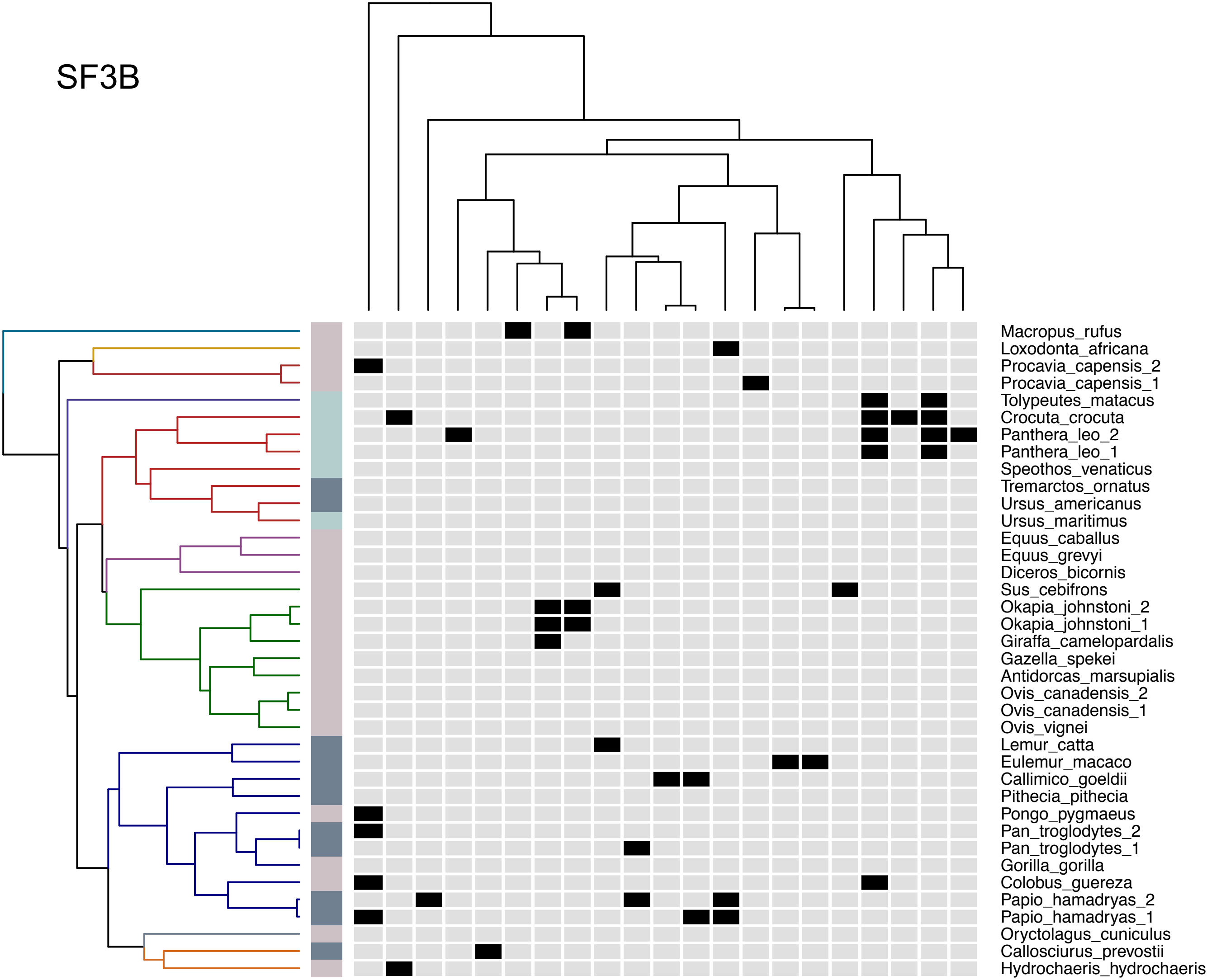
SF3B
Macropus_rufus
Loxodonta_africana
Procavia_capensis_2
Procavia_capensis_1
Tolypeutes_matacus
Crocuta_crocuta
Panthera_leo_2
Panthera_leo_1
Speothos_venaticus
Tremarctos_ornatus
Ursus_americanus
Ursus_maritimus
Equus_caballus
Equus_grevyi
Diceros_bicornis
Sus_cebifrons
Okapia_johnstoni_2
Okapia_johnstoni_1
Giraffa_camelopardalis
Gazella_spekei
Antidorcas_marsupialis
Ovis_canadensis_2
Ovis_canadensis_1
Ovis_vignei
Lemur_catta
Eulemur_macaco
Callimico_goeldii
Pithecia_pithecia
Pongo_pygmaeus
Pan_troglodytes_2
Pan_troglodytes_1
Gorilla_gorilla
Colobus_guereza
Papio_hamadryas_2
Papio_hamadryas_1
Oryctolagus_cuniculus
Callosciurus_prevostii
Hydrochaeris_hydrochaeris

SF3C

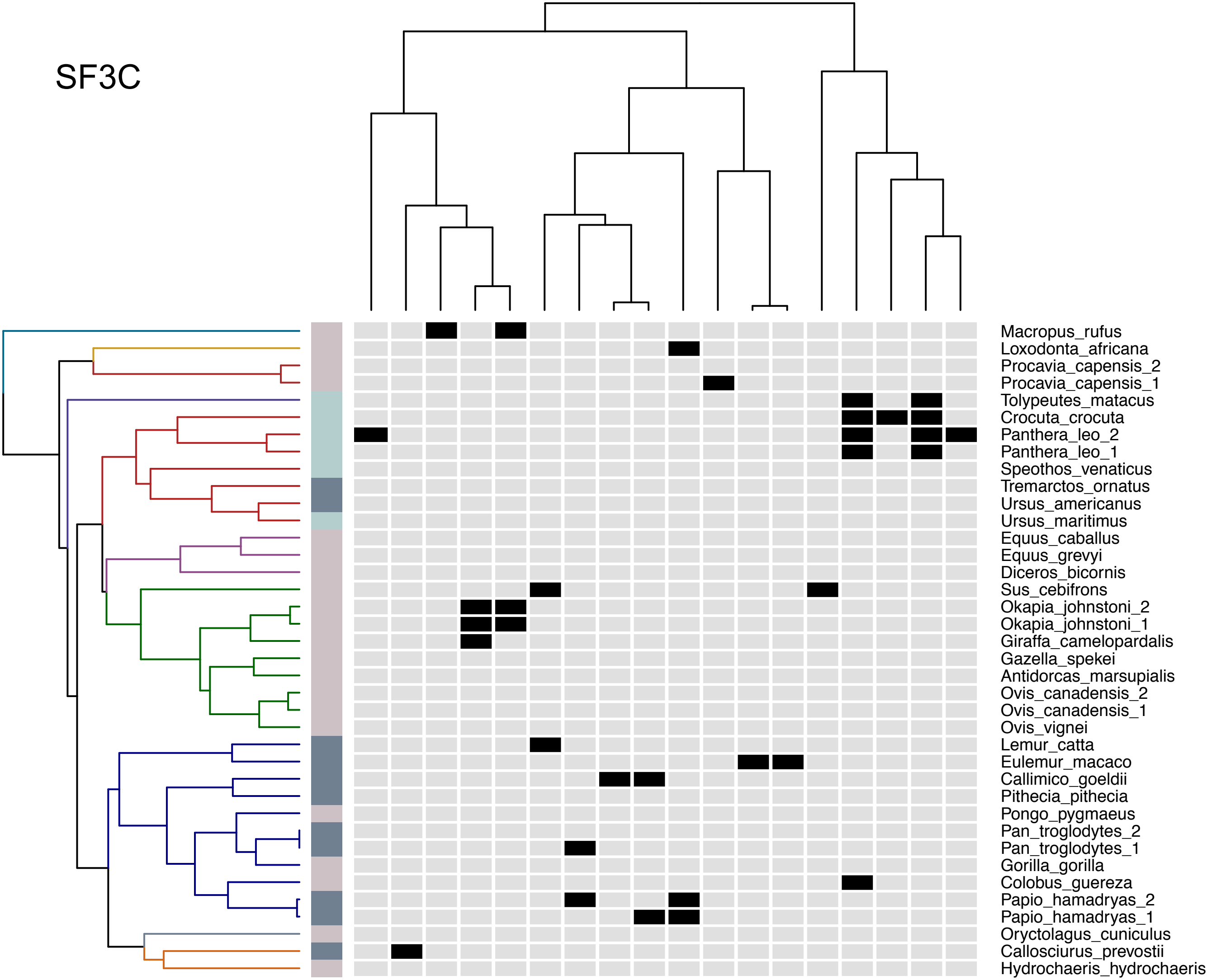
Macropus_rufus
Loxodonta_africana
Procavia_capensis_2
Procavia_capensis_1
Tolypeutes_matacus
Crocuta_crocuta
Panthera_leo_2
Panthera_leo_1
Speothos_venaticus
Tremarctos_ornatus
Ursus_americanus
Ursus_maritimus
Equus_caballus
Equus_grevyi
Diceros_bicornis
Sus_cebifrons
Okapia_johnstoni_2
Okapia_johnstoni_1
Giraffa_camelopardalis
Gazella_spekei
Antidorcas_marsupialis
Ovis_canadensis_2
Ovis_canadensis_1
Ovis_vignei
Lemur_catta
Eulemur_macaco
Callimico_goeldii
Pithecia_pithecia
Pongo_pygmaeus
Pan_troglodytes_2
Pan_troglodytes_1
Gorilla_gorilla
Colobus_guereza
Papio_hamadryas_2
Papio_hamadryas_1
Oryctolagus_cuniculus
Callosciurus_prevostii
Hydrochaeris_hydrochaeris

SF3D

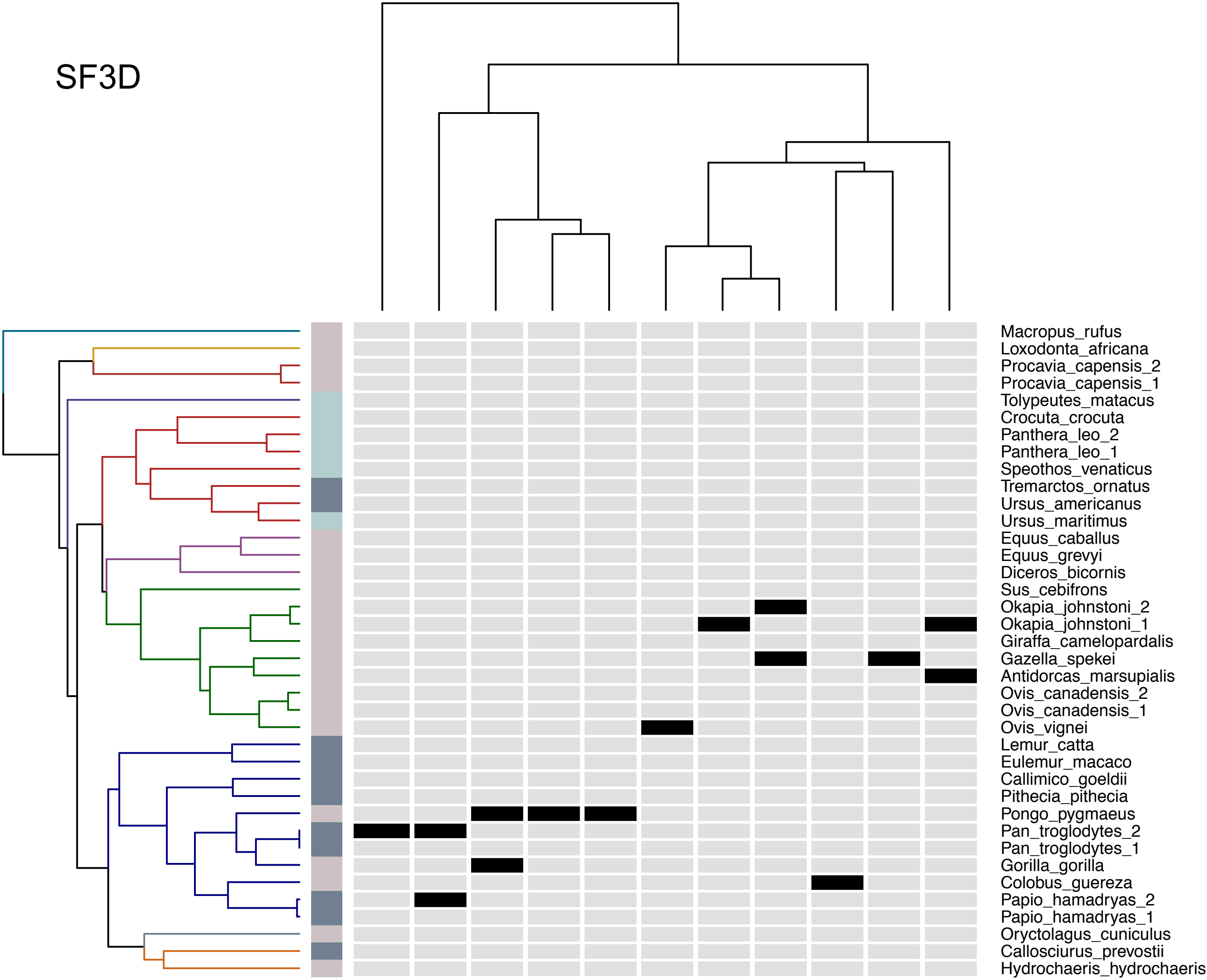
Macropus_rufus
Loxodonta_africana
Procavia_capensis_2
Procavia_capensis_1
Tolypeutes_matacus
Crocuta_crocuta
Panthera_leo_2
Panthera_leo_1
Speothos_venaticus
Tremarctos_ornatus
Ursus_americanus
Ursus_maritimus
Equus_caballus
Equus_grevyi
Diceros_bicornis
Sus_cebifrons
Okapia_johnstoni_2
Okapia_johnstoni_1
Giraffa_camelopardalis
Gazella_spekei
Antidorcas_marsupialis
Ovis_canadensis_2
Ovis_canadensis_1
Ovis_vignei
Lemur_catta
Eulemur_macaco
Callimico_goeldii
Pithecia_pithecia
Pongo_pygmaeus
Pan_troglodytes_2
Pan_troglodytes_1
Gorilla_gorilla
Colobus_guereza
Papio_hamadryas_2
Papio_hamadryas_1
Oryctolagus_cuniculus
Callosciurus_prevostii
Hydrochaeris_hydrochaeris
