# Supplementary material for: Ecophylogenetics Clarifies the Evolutionary Association between Mammals and Their Gut Microbiota
Source: mBio. 2018 Sep 11;9(5):e01348-18. doi: 10.1128/mBio.01348-18 (PMC6134092; doi:10.1128/mBio.01348-18)
Supplement: TEXT S2 [file mbo004184055s2.pdf]

Assessing the Sensitivity of Phylogenetic Reconstruction to Sequence Clustering Methods

We analyzed samples obtained from thirty different hominids that were interrogated as part of the Moeller et al. analysis, clustered their respective 16S sequence data using QIIME and DADA2, and measured how phylogenies assembled by each clustering procedure differed. We restricted our analysis to 10 randomly selected individuals from each of the following hominid species: gorillas, chimpanzees and bonobos. Humans were not selected because the corresponding public data did not include quality scores, which is necessary for DADA2. We clustered sequences using the same QIIME workflow as articulated in the main text. In parallel, we followed the developer-recommended DADA2 (v.1.8) workflow that is outlined in their github repository (https://benjjneb.github.io/dada2/tutorial.html). Importantly, these two workflows use different sequence alignment procedures, with QIIME leveraging PyNAST while DADA2 used Maffit. As shown below, despite these differences in alignment procedure, which could result in significant variation in the subsequence FastTree phylogenies that we assembled, the correlation between trees remained high.

QIIME produced 19,299 OTUs after chimera filtering, while DADA2 produced 3,884 OTUs. Prior to multiple sequence alignment and phylogenetic reconstruction, QIIME sequences were trimmed to 240 bp to match those produced by DADA2's quality trimming. For each data set, *de novo* phylogenetic trees consisting of 19,299 and 3,884 tips were constructed from the trimmed sequences using FastTree (*FastTree-2.1.10 –nt -gtr*). It is challenging to directly compare the results of independent clustering analyses, since the number of clusters and the membership of clusters will vary across analyses. To solve this problem, we identified all shared OTU reference sequences between QIIME and DADA2 and restricted our analyses to these overlapping sequences (i.e., all non-identical sequences were pruned from each tree). This decision is justifiable because the purpose of our analysis is to determine how the different clustering procedures affect the phylogenetic relationship of the lineages included in the tree, as opposed to determining how QIIME and DADA2 impact the composition of each cluster. The resulting phylogenies contained 2,293 lineages, which we treated as a representative sample of lineages to answer the aforementioned question.

The cophenetic function in the vegan R package quantified the phylogenetic distance between all pairs of lineages in each tree. A mantel test found that these pairwise phylogenetic distances were strongly correlated across the two clustering methods (Mantel $R^2 = 0.6688$, $p < 0.001$). The correlation substantially improved when two outlier lineages, which were identified as taxa whose mean pairwise distance were greater than the 95% confidence interval of average distances on either tree, were dropped from the analysis (Mantel $R^2 = 0.7615$, $p < 0.001$; Figure 1). We also plotted the two phylogenies to assess their topological concordance. As illustrated in Figure 2, there are few topological differences between the QIIME and DADA2 phylogenies and topological variation tends to retain the local topological structure of the subtending taxa.

Collectively, these results demonstrate that QIIME and DADA2 tend to produce highly correlated phylogenies and that the analysis of a phylogeny produced using QIIME clustered data will tend to reflect the phylogeny produced using the DADA2 clustered data.

Supporting Figures

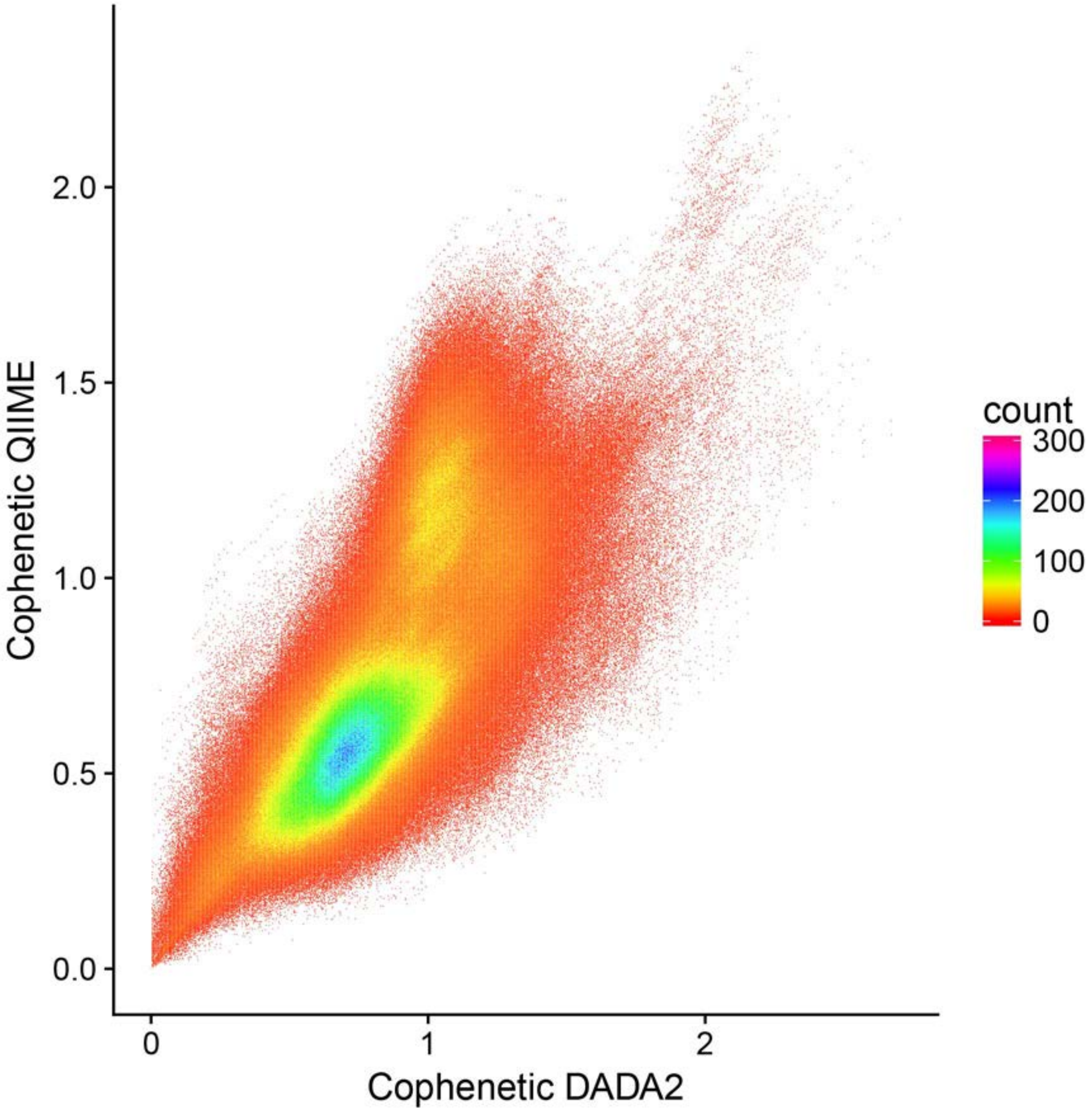

Figure 1: Scatterplot of the pairwise distances spanning lineages found in both the QIIME and DADA2 clustered 16S sequence phylogenies. Each point in the scatterplot represents a pair of lineages found in a phylogeny. The x-axis indicates the phylogenetic distance for a pair of lineages as measured in the DADA2 tree while the y-axis indicates the phylogenetic distance for the same pair of lineages as measured in the QIIME tree. The color of the point represents the density of pairs plotted at any given point.

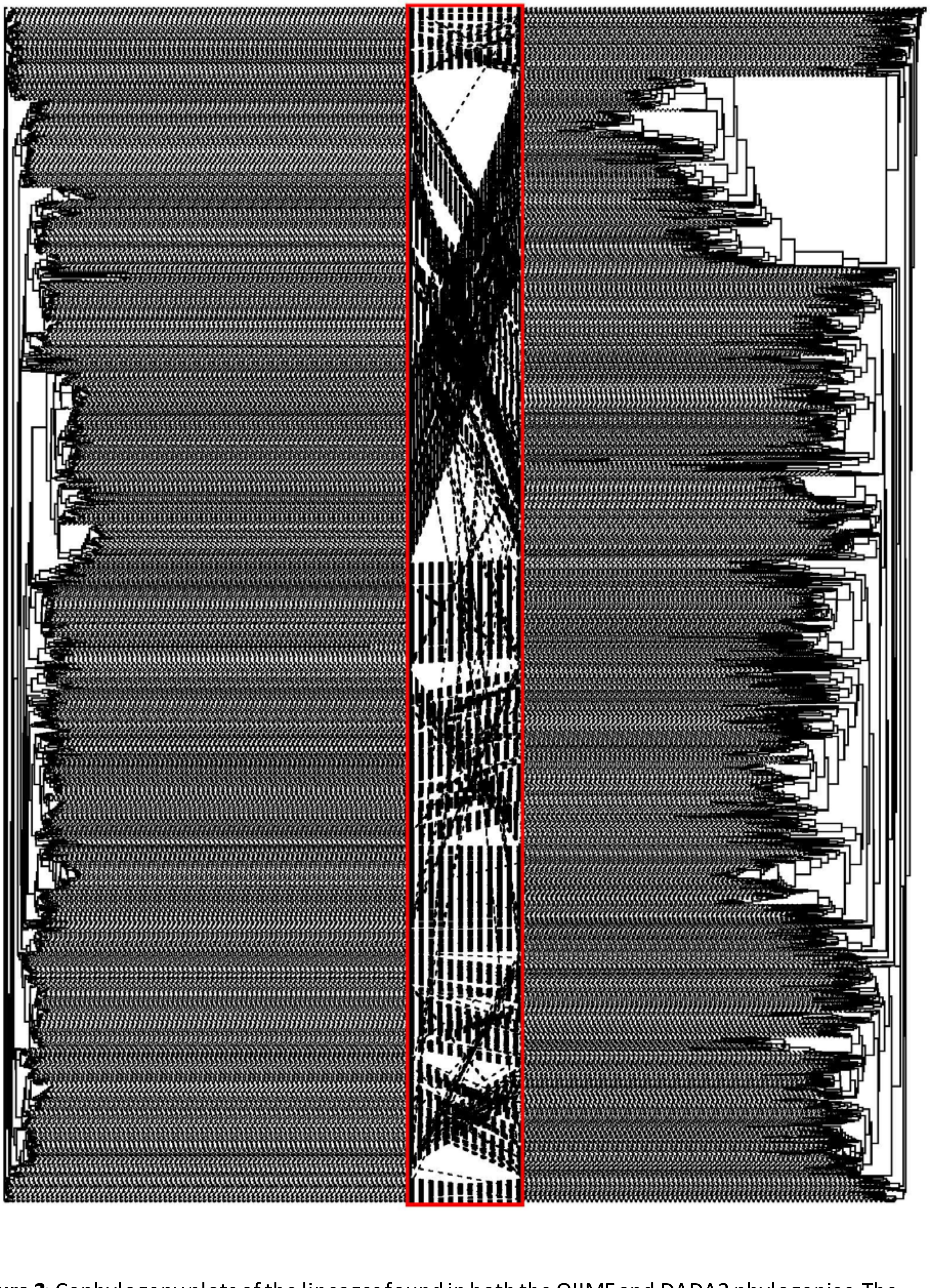

Figure 2: Cophylogeny plots of the lineages found in both the QIIME and DADA2 phylogenies. The phytools::cophylo function in R (with node rotation) compared illustrates the DADA2 phylogeny on the

left and the QIIME phylogeny on the right. The center column of thicker dotted lines indicates how tips of identical names map between the two phylogenies. Fine-dotted lines are not informative, but rather serve to indicate which lineage corresponds to which of the center column lines.
